# Barriers and facilitators to education access for marginalised non-citizen children in Malaysia: A qualitative study

**DOI:** 10.1371/journal.pone.0286793

**Published:** 2023-06-02

**Authors:** Tharani Loganathan, Zhen Ling Ong, Fikri Hassan, Zhie X. Chan, Hazreen Abdul Majid

**Affiliations:** 1 Centre for Epidemiology and Evidence-based Practice, Department of Social and Preventive Medicine, University of Malaya, Kuala Lumpur, Malaysia; 2 Department of Global Health and Development, Faculty of Public Health and Policy, London School of Hygiene and Tropical Medicine, London, United Kingdom; 3 Centre for Population Health, Department of Social and Preventive Medicine, University of Malaya, Kuala Lumpur, Malaysia; 4 School of Chiropractor, AECC University College, Parkwood Campus, Bournemouth, Dorset, United Kingdom; Caleb University, NIGERIA

## Abstract

In Malaysia, marginalised non-citizen children are excluded from formal education at public schools. Recognising education as a fundamental human right, the barriers and facilitators to educational access among refugee and asylum-seeker, migrant, stateless and undocumented children in Malaysia were explored. Qualitative data were collected via 32 in-depth interviews with multiple stakeholders. Data were thematically analysed and organised at three socio-ecological levels. At the ‘legislative and policy’ level, the requirement of citizenship documents only allows some stateless children to access public schools. Yet, many informal learning centres are not state-endorsed, as they are unable to fulfil licensing criteria. Importantly, denying the right to work for adult refugees and other undocumented people demotivates the pursuit of education among children. At the ‘individual and family’ level, financial constraints constitute a major reason for school dropouts, especially through expectations on boys to work. Cultural norms partly contribute to the lower enrolment of Rohingya refugee girls in secondary education, but gender parity is maintained for most in primary education. Another factor is proximity to learning centres, which links to safety concerns and transportation costs. Those who accessed public schools reported bullying by local children, which parallels institutional discrimination against marginalised non-citizens. At the ‘community and educational institutions’ level, inadequate funding for learning centres limits their ability to invest in physical facilities, teachers’ salaries and others. Despite difficult operating conditions, learning centres address diverse school readiness, educational backgrounds, and language competencies among students by having sensitised teachers, placement tests and preparatory classes at school entry, and options for vocational training. We propose the gradual inclusion of all children in public schools and the immediate state recognition and support of learning centres. Correspondingly, realising the ‘Right to Work’ for refugees and stateless peoples will be synergistic in advancing universal education access for all children.

## Introduction

Education is a fundamental human right [1], integral in enabling marginalised children and their families to achieve socioeconomic stability [2–4]. Yet, the average primary school completion rate worldwide is only 90% in 2020 [5]. In Malaysia, primary education is compulsory for all Malaysian children aged from 6 to 12 years, and the country has achieved almost universal primary and lower secondary enrolment rates [6–9]. However, in 2015, an estimated 100,000 to 250,000 children were found to be not enrolled in primary and secondary schools respectively [10].

Most vulnerable to educational deprivation in Malaysia are non-citizen children considered marginalised including refugees and asylum-seekers in Peninsula Malaysia, children of migrant workers, and stateless and undocumented persons in the state of Sabah in East Malaysia. Malaysia has not ratified the 1951 Convention on the Status of Refugees and its 1967 Protocol, and the 1954 Convention on the Status of Stateless Persons [11–13]. As such, refugees, asylum seekers, and stateless persons are treated similarly to undocumented migrants, with limited entitlements to education, healthcare, and formal employment [14–16].

Non-citizens in Malaysia were estimated at 2.7 million in 2021, of which 472,200 were children aged from 0 to 19 years [17]. The majority of refugees in Malaysia are stateless Rohingyas and other ethnic groups from Myanmar, while others are from 50 different countries [18]. Of the 23,823 refugees and asylum-seeking children of school-going age registered at UNHCR Malaysia, only a third were enrolled in learning centres, and a small number [874 (16%)] were enrolled in secondary education [19, 20]. Migrant children refer to children of low-skilled economic migrants, who are likely to be undocumented as immigration laws prohibit migrant workers from bringing dependents or from having children in Malaysia [21]. The state of Sabah hosts diverse non-citizen groups, due to intergenerational cross-border migration with neighbouring Philippines and Indonesia. Non-citizens in Sabah constitute up to a third of the population (995,400), of which about 250,000 are children aged from 0 to 19 years [22, 23]. Children at risk of educational exclusion in Sabah include children of Filipino and Indonesian migrants, the stateless Bajau Laut tribes and indigenous people facing geographical and other barriers to birth registration.

In Malaysia, education provision is governed by the Education Act 1996 (Act 550). Reservations made in Article 28 of the Convention of the Rights of the Child (CRC) of which Malaysia is a signatory [24, 25], and amendments to the Education Act 1996 (Act 550) have omitted non-citizen children from the nation’s commitments towards universal education, placing them at high risk of education exclusion [26]. Unable to enter public schools, marginalised non-citizens have limited access to formal education [27]. Since private education is too expensive to be a viable option, marginalised non-citizen children are reliant on informal education from alternative or community learning centres supported by civil society organisations, faith-based organisations, private donors, and local communities [28–30].

The often ‘undocumented’ status of marginalised non-citizen children means that many are statistically invisible. This makes it challenging to measure the nation’s progress towards achieving UN Sustainable Development Goal Target 4.1—universal access to free, equitable and quality primary and secondary education. To advance the right to education for all, this study aims to understand the barriers and facilitators to educational access faced by marginalised non-citizen children in Malaysia, through in-depth interviews of multiple stakeholders.

## Materials and methods

### Study design

We used qualitative methods to explore and better understand the barriers and facilitators to education access faced by marginalised non-citizen children in Malaysia. Marginalised non-citizen children refer to refugee and asylum seekers, migrant, stateless and undocumented children in Malaysia. International students and children of expatriates were excluded from this study.

### Definition of terms

Refugees are those forcibly displaced from their country of nationality or usual residence and are unable or unwilling to return due to a well-founded fear of persecution [11, 31]. Refugees are recognised and protected by international law. Asylum-seekers are individuals seeking international protection, but whose refugee status has yet to be officially determined. Migrant children refer to children of low-skilled migrant workers. Since immigration laws and employment contracts prohibit migrant workers from bringing dependents or from having children in Malaysia, migrant children are likely to have unregistered births and to be undocumented. According to the 1954 Convention relating to the Status of Stateless Persons, stateless persons are not considered as nationals by any State under the operation of its law and this includes persons with undetermined nationality [12]. The term undocumented persons refer to anyone residing in the country without legal documentation, including people who entered the country without valid passports or permits, and children without birth certificates.

### Sampling and recruitment

Non-citizen groups in Malaysia are heterogenous, with different privileges and opportunities for education [30]. We aimed to interview different stakeholders to better triangulate the barriers and facilitators faced by non-citizen children in seeking access to education in Malaysia. Participants were recruited to represent the diversity of experiences of non-citizen populations in Malaysia, specifically the (1) stateless and undocumented children applicable to all of Malaysia, (2) refugees and asylum-seekers in Peninsular Malaysia and (3) migrant, stateless and undocumented children in Sabah, East Malaysia.

We purposely sampled key informants with experience in non-citizen education in Malaysia, including (1) community organisers from civil society organisations, (2) former students that were adult refugees with experience as children navigating the education system in Malaysia, (3) education providers from learning centres and schools, (4) policymakers from government and international organisations, and (5) academic researchers with professional expertise in the education of refugee, stateless and migrant populations in Malaysia.

Potential participants were identified during an initial desk-based review and through the research team’s local knowledge of key actors in non-citizen education. We created a database of potential study participants according to their professional backgrounds and experience with non-citizen population groups in Malaysia. We then initiated contact with these participants via emails, phone calls or messages through LinkedIn and other social media platforms. Additional participants were recruited via snowball sampling. Potential participants were sent participant information sheets and consent forms. Recruitment stopped when researchers agreed that further interviews would not yield additional information.

### Data collection

Semi-structured interview guides were initially developed based on a desk review we conducted to understand non-citizens education policies in Malaysia, between June 2020 to June 2021. The interview guides contained introductory questions to understand participants’ backgrounds and to contextualise different non-citizen groups. Open questions were asked on (a) knowledge and experiences education policy and services for non-citizens, (b) perceptions or experience with non-citizen access to education, (c) experiences of challenges and facilitators for education access and (d) suggestions for the improvement in education policy or services for non-citizens. These guides were developed for three main categories of interviewees: (a) teachers and educators, (b) community organisers and migrant representatives, and (c)policymakers and high-level stakeholders. We customised interviews according to the background of the interviewee. Minor revisions were made to the guide after initial reflections from the earlier interviews, see S1 File for interview guides.

In-depth interviews were conducted from June 2020 to March 2021. In total, we conducted 32 in-depth interviews with 33 individuals. Most interviews were conducted on an individual basis. However, two interviews were conducted with 2 participants from the same organisation. One participant was interviewed twice.

Due to movement restrictions during the COVID-19 pandemic, most interviews (n = 28) were conducted remotely using an online platform (Microsoft Teams), while 5 interviews were conducted in person in Kuala Lumpur, Malaysia. Interviews ranged from 1 to 1.5 hours in length and were conducted either in English or Bahasa Malaysia (Malay language) according to participants’ preferences. Audio recordings were transcribed verbatim. Concurrent analysis during data collection informed the further refinement of question guides.

We interviewed community organisers from civil society organisations who were representatives of non-citizen communities, and/or were representatives of civil society organisations that supported learning centres. The former students interviewed were from different refugee communities living in Peninsular Malaysia, including the Rohingya and Chin communities from Myanmar, Syrian, Ahmadiyya communities from Pakistan, Somalian and others. Several of the community organisers interviewed were also members of the refugee community and were former students in Malaysia. The education providers interviewed were teachers at learning centres and schools catering to non-citizen populations. Most of the education providers interviewed also identified themselves as community organisers. We interviewed policymakers and government officials involved in non-citizen children’s education from Malaysia and neighbouring countries, and representatives of international organisations based in Malaysia. Academic researchers interviewed had varied expertise on the education and legal entitlements of refugee, stateless and migrant populations in Malaysia. The characteristics of the study participants are shown in Table 1.

**Table 1:** Characteristics of the study participants (n = 33).

**Participants’ primary role**	** **	**Label**	**No.**
Community organiser		CO	4
Former students^1^		FS	7
Education provider^2^		EP	11
Policymaker		POL	4
Researcher		RES	7
**Total**	** **	** **	**33**
**Non-citizen type**	**Peninsular Malaysia**	**Sabah**	**Overall Malaysia**
Overall—non-citizens			5
Refugees	15		
Stateless	3	4	2
Migrant^3^		3	1
**Total**	**18**	**7**	**8**

^1^All the former students interviewed were adult refugees. Of the 7 interviewed, 3 were also education providers.

^2^ Of the 11 education providers interviewed, 7 also identified themselves as community organisers.

### Data analysis

Data analysis was conducted in an immersive, exploratory, and inductive manner, with regular discussions between researchers to refine codes and identify new themes. Thematic analysis was conducted as described by Braun and Clarke, where themes were identified and reported using six phases: (1) becoming familiar with the data, (2) generating initial codes, (3) searching for themes, (4) reviewing themes, (5) defining themes, and (6) producing the report [32]. The main themes were organised according to barriers identified at different socio-ecological levels (‘legislative and policy’, ‘individual and family’, and ‘community and education institutions’) and subsequently analysed based on a conceptual framework of access to education, adapted from Levesque et al.’s work on healthcare access [33].

To identify our themes, two authors (TL and ZC) conducted detailed coding of transcripts. Codes and themes were refined by repeated readings of transcripts, field notes and impressions. Regular bi-weekly discussions among the research team (TL, ZO, FH, ZC and HM) ensured consistency in coding, resolution of disputes and allowed further refinement of coding and collapsing codes into themes, in addition to giving attention to negative cases and minor themes. Interviews with stakeholders from different backgrounds allowed for the triangulation of findings.

Transcripts were coded into emerging themes using NVivo 12 Plus, (QSR International, Melbourne, Australia) and quotations were extracted into Microsoft® Excel® for Office 365, (Microsoft, Redmond, WA, USA). Interviews in Bahasa Malaysia were analysed in the same language, while extracted quotations were translated to English for publication.

### Reflexivity

Interviews were conducted by a team of medical doctors and academic researchers, who could be perceived as trusted authority figures. To counter possible power imbalances, especially among vulnerable non-citizens, participants chose interview times and locations. The research team strongly valued the rights of all humans to education but did not allow this to drive participant sampling (i.e., we did not sample participants based on their views but by their subject matter experience), nor impose our beliefs on study participants, allowing unbiased sharing.

### Ethics

For ethical reasons, we chose not to interview children. All study participants were adults and were able to provide informed consent. Participant information sheets were distributed and written informed consent was obtained from all participants before commencement of interviews. Study participation was voluntary, and participants were informed that they were free to refuse to answer questions or terminate interviews at any point. All participants agreed to be audio recorded and quoted anonymously in publications. Audio recordings and electronic transcripts were stored in secure data servers. This study was approved by the Medical Ethics Committee, University Malaya Medical Center (Approval numbers: UM.TNC 2/UMREC).

## Results

### Conceptual framework

**Fig 1:**
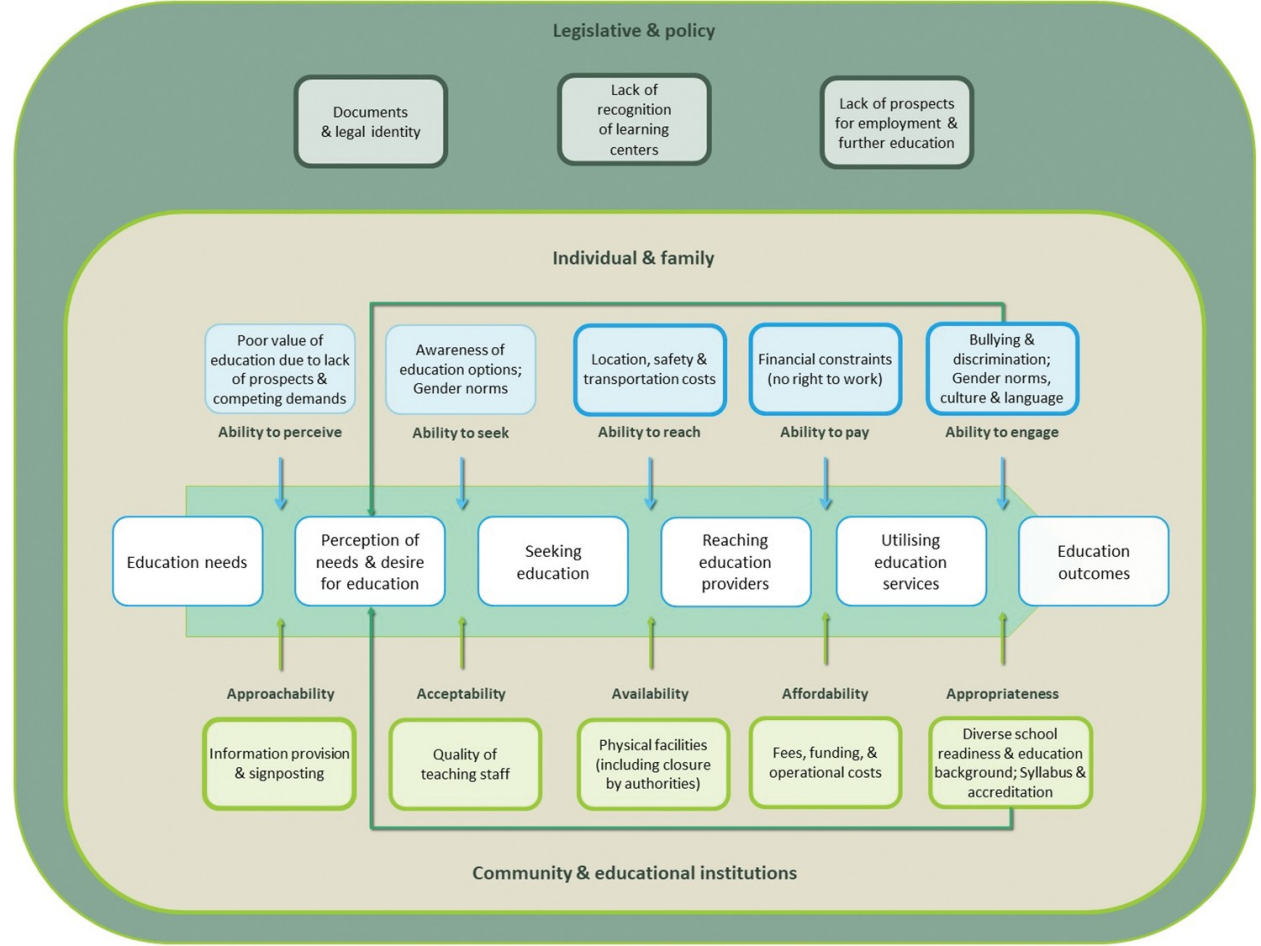
Conceptual framework of barriers to education access for non-citizen children in Malaysia. This framework was adapted from Levesque et al.’s framework on healthcare access [33]. While all factors listed are grounded in the interview data, boxes with bold outlines indicate major themes from the qualitative thematic analysis.

The barriers identified were organised into three socio-ecological levels and a conceptual framework of education access adapted from Levesque et al.’s work on healthcare access [33] (Fig 1). Education access is defined here as the opportunity to have educational needs fulfilled; it constitutes demand-side (individual and family) and supply-side (community and educational institutions) determinants along the process of perceiving education needs, desiring, seeking, reaching, utilising, and benefiting from education services. Five dimensions of ability to realise access (ability to perceive; seek; reach; pay; engage) correspond to five provider dimensions of service accessibility (approachability; acceptability; availability; affordability; appropriateness). At the top, ‘legislative and policy’ level determinants influence how barriers to education manifest at lower ecological levels—supply and demand. The looping arrow represents how education access is not a linear process and that the five dimensions are interlinked. For instance, a syllabus that does not cater to the needs of refugee communities intending to resettle in an English-speaking country, or negative experiences of bullying among some non-citizens who accessed public schools could decrease the desire for education and lead to school dropout.

While this study focuses on barriers, we have identified and included some strategies and adaptations by community and educational institutions that serve as facilitators under these challenging operating conditions. Study results are presented by socio-ecological levels: ‘legislative and policy’, ‘individual and family’, ‘community and educational institutions’.

Table 2 summarises the major barriers and facilitators of non-citizen children’s access to education identified in this study.

**Table 2:** Barriers and facilitators of non-citizen children’s access to education.

	Barriers	Facilitators
**Legislative and policy**	• Lack of identity documents excludes non-citizen children from public education • Learning centres face difficulties with registration and are a risk of shutdown due to unlicensed operations • Lack of prospects for employment and further education for undocumented persons, including refugees	• Learning centres do not require legal documents for enrolment • UNHCR protection letter to allow operation of learning centres for refugees • Options for international school-leaving examinations in preparation for resettlement
**Individual and Family**	• Lack of the right to work leads to financial hardship and increased school dropouts • Safety concerns and transportation costs • Bullying and discrimination from local children and teachers • Gender norms, culture and language	• Borrowing money from the community, part-time work and private sponsorship • Provision of transportation and proximity of learning centres to communities • Learning local languages and the ability to blend in by adopting a more ‘Malaysian’ identity • Early countermeasures applied to sensitize children on cultural differences
**Community and Educational Institution**	• Inadequate information on available education options • Funding constraints limit the hiring of trained teachers and explain the reliance on volunteers • Lack of standardization of syllabus and accreditation of school-leaving examinations • Inadequate physical facilities and infrastructure • Fees, funding, and operational costs Diverse school readiness and educational backgrounds	• Dissemination of information on available learning centres among the community • Investment in teachers’ training and salaries • Availability of MOE teaching materials, flexible syllabus, and vocational training • Financial support from the government, embassies, employers & philanthropists • Rapid assessment, placement tests and catch-up programmes

Facilitators are noticeably clustered at lower socio-ecological levels and are insufficient to redress significant structural barriers. The presence and sufficiency of these facilitators vary greatly across non-citizen subgroups and education providers.

### Main results

#### Legislative and policy

*Documents and legal identity*. The Zero Reject Policy was launched in 2019 to allow stateless children access to primary-level education at public schools. This policy was described as restrictive as only children with at least one Malaysian parent and those in the process of applying for Malaysian citizenship are allowed to enrol in public schools. Undocumented, stateless, refugee or migrant children born to non-citizens are not allowed entry to public schools. Participants explained that public schools frequently rejected non-citizen children’s applications for enrolment based on incomplete legal and identity documents.

*“As of last year January [2019], the government policy is that all stateless children can go to school. But the actual implementation process is a little different. Because you still need a birth certificate to go to a government school, you still need a marriage certificate to go to school. Only stateless children can go to school—where the definition of stateless is—one parent is Malaysian, and one parent is not. Refugee children cannot go to government schools. They have no access. And in many cases, what we noticed at the beginning of last year, was that many children went to apply to go to school and were kicked out within the first two weeks.”CO-02*

Study participants also informed that it was more difficult for children with incomplete documentation to enrol in public schools, as the new policy had introduced additional bureaucracy that prevented students from entering at the discretion of school heads.

Unlike public schools, most learning centres do not require any legal or identity documents for enrolment. Refugee learning centres that receive support from UNHCR may require some proof that the children have registered or are in the process of registering with the UNHCR before enrolment into the learning centre. However, respondents explained that asking for documentation may deter marginalised groups from enrolling their children at learning centres and has thus not been made a requirement.

*“In the beginning, we said, ‘You must have a UN card or a UN letter’. And the moment we open our mouths to ask for this [document] some parents would just move away. So now, we just take everybody, whether they have a paper or no paper. I think children need education regardless, it is their right to have some form of education whether they have papers or not, and regardless of their legal status. So that is the stand that we have taken.” EP-08*

One education provider shared that since document checks aren’t imposed as an entrance criterion, refugee learning centres may inadvertently accept migrants and other non-citizen children.

*Lack of recognition of learning centres*. While only certain non-citizen children are allowed into public schools, the government has permitted the establishment of an alternative education system through learning centres run by NGOs and other stakeholders. The registration of learning centres is under the private education development sector of the Ministry of Education (MOE) and the criteria for registration have been described as rigorous, and include the minimum standards for teachers’ qualifications, syllabus, and specifications for safety and physical facilities.

Participants informed that learning centres lack the resources to fulfil the stringent criteria set by the MOE. Nevertheless, individuals and civil society organisations continue operations at learning centres despite not having a formal endorsement from the MOE. This education provider felt that it was unjust for government authorities to impose unrealistic expectations on NGOs with limited resources.

*“They wave the manual in front of me and said*, *‘You have to follow this and that*!*’ You know the big thick documentation*, *framed by MOE about how to set up schools*. *That is ridiculous… absolutely ridiculous*! *Our schools have nothing to do with that at all*. *We cannot meet a single criterion in it*. *Our schools are very poor–we have no budget; we have no income*! *We do not have a land area—we just rent*, *or we just squat somewhere*. *There isn’t a single thing in that documentation that we can meet*. *But for us*, *the most important thing is educating children*, *so they don’t become social problems*, *right*? *So*, *even though we cannot follow any of the ‘Malaysian school’ rulings*, *we will still carry on with our schools because we believe it is very important that children have a future*, *and every child has the opportunity to have access to education and health facilities*.*”* EP—04

Education providers and community organisers, mainly from Sabah, shared that unlicensed learning centres were regularly shut down and were ordered to pay hefty fines. In contrast, refugee learning centres registered with the UNHCR in Peninsular Malaysia are offered some leeway to operate by local authorities.

*Lack of prospects for employment and further education*. The legislative framework in Malaysia does not allow for the formal employment of undocumented persons including refugees. Interviewees shared that without employability, children lack the motivation to pursue education beyond basic skills and competencies.

*“For a lot of them, right? So, you cannot really work, you cannot really get a proper job. So, we notice this, a lot of them do go to school because they want to learn how to have basic reading, writing, and arithmetic so that they can use money in the market to help sell things. And then they would drop out because they feel that this education will only help me to this point, [and] because the chances of me going to university and doing things [after university] are very remote. That [idea of] ‘pursuing education for education’ is something so remote that they do not see that link and relevance.” POL-02*

This policymaker explained that education providers need to be more realistic in their encouragement of education for non-citizens.

Interviewees shared examples of refugees with high qualifications, forced to settle for low-wage jobs without formal contracts, and are at risk of exploitation.

*“To be honest, jobwise [and] the future is like super terrible! This is just a very honest and straightforward statement that jobwise it is like super, super horrible. My father is a doctor by profession, but he is working as a security guard [here]. His job is not confirmed, some days he is not called [up to work]. So, he will not go. Just like a daily wage worker. All the jobs that refugees are offered in Malaysia, are just labour jobs or daily wage jobs… you have it one day, but another day you don’t have it. And the other challenge that comes with it is the salary, sometimes you will not be paid at all. The boss will be like, ‘Oh, no! no! Go away, we do not need you at all! You have a refugee status!’ FS-05*

Non-citizen children are not allowed to sit for local Malaysian school-leaving examinations. Refugee children may sit for international examinations (IGSCE or the British O-Levels) in preparation for future resettlement in third countries. However, few children pursue international examinations conditional on the availability of private sponsorship for the prohibitive examination fees. Interviewees also question the value of obtaining school leaving certificates, as non-citizen children have limited options for tertiary education and job prospects in Malaysia.

#### Individual and family

*Financial constraints*. Communities living in poverty have livelihood concerns. Interviewees informed that many marginalised children were forced to drop out of school to enter the workforce as their family’s immediate financial needs were more critical compared to education.

*“In my experience*, *people who live in poverty and people who are poor*, *what they can think is only about finding money and working for their daily basic needs*. *And they would not encourage their children*, *even though the children are really smart and very good at learning*. *Um*, *they are not really supportive and that is really sad*! *Actually*.*”* FS-04

Dropping out of school at post-primary levels is an economic reality faced by non-citizen children, as adults are denied formal employment. This respondent shared his experience of dropping out of a learning centre as an adolescent because he could not cope with the additional pressures of working.

*“It was really going very well for almost a year*. *But during that year*, *in the morning I would go to school*, *and in the afternoon when I finish*, *I will go home and then straight away go to the restaurant to work [the] night shifts*. *And then again*, *I wake up in the morning and head to school*. *So*, *I missed a lot of classes and I could not do much of my homework*. *I could not study very well because I was constantly very tired from work at the restaurant*. *And I was very young*, *I was only 13 to 14 years old at that time*. *I could not really focus on my school only*, *I had to focus on my work as well because I wanted to support my family*. *And so*, *that’s when I decided to stop going to school and continue working instead*.*”* FS-03

Education providers explained that community engagement is crucial to ensure students facing financial distress avoid dropping out of school. Learning centres also waive fees for students facing financial hardship.

*“Our teachers work very closely with the community and the parents especially. We have teachers that do home visitation. So, if students do not come to school for three days and above, they would get a home visit. And if they do not pay fees and or if they have any signs of distress, teachers will be looking out for them. So, we have teachers [and] we have a team of social workers too that goes to the community and stays quite close with most of our students. We actually keep quite good track [of them].” EP-08*

*Location*, *safety*, *and transportation costs*. Education providers informed that safety while travelling and the costs of transportation are concerns for parents of school-going children.

Most study participants concurred that the non-citizen community regularly face harassment and extortion by authorities on the premise of examining legal identity documents. This education provider explained that in addition to the fear of harassment, non-citizen parents are also concerned about human traffickers targeting marginalised children, as they have little legal recourse in Malaysia.

*“As you know, the children, when they go from home to school, they would have to walk a certain distance. And a lot of parents’ fear harassment from local authorities. And even from gangsters in the area, they do not have protection in that sense, right. So, first is the local authorities, right? Because in terms of harassment, local authorities actually extort money and are harassing…. The fear has stopped parents from sending their children to school. They were very scared that the children would be snatched away, if not by local authorities, then by gangs in that area that we’re dealing with child trafficking, [or] child prostitution.” EP-01*

The UNHCR card provides some protection for refugees against immigration detention. As such, refugees and asylum-seekers face increased vulnerability when their UNHCR card is not renewed or their refugee status is not verified, and this would affect school attendance.

*“There would be security issues, then they are also likely to be pulled out of school, right. If suddenly their family loses their UNHCR card status, right. Then it becomes dangerous, especially since the cops like to wait at certain points and bug them. This is also a particular reason for them to stop schooling sometimes. But it does affect their attendance in class because once there is a whisper of an ‘operasi’ [immigration operation]. Those without documentation either stop coming for a while or completely drop out and that does affect attendance.” EP-07*

Education providers interviewed have realised that it is beneficial to locate schools near or within communities to enable easy access to learning. Others provide free transportation for their students, both to subsidise costs and for students’ safety.

*“Transportation is a big issue and is expensive in Malaysia*, *you know*. *So*, *how do we move students from here to there*? *For example*, *I cannot tell the refugee of 8- [or]*, *9-year-olds*, *‘Okay*, *please take the bus and then go down to the LRT (Light Rail Transport)*, *take the LRT here [and] there’ and all that–I mean*, *they need to be secure*! *Child protection is something that is very important*. *We all need to be very*, *very careful about how we operate*.*”* EP-03

*Bullying and discrimination*. Under the current education policy, only stateless children with Malaysian parents are allowed to enter public schools. In the past, refugee children had participated in public school education at the discretion of school heads. Non-citizen children may also enter religious schools together with Malaysian children. Former students and education providers interviewed informed that bullying experienced by non-citizen children was mainly from local children.

Rohingya refugees and the stateless peoples of Sabah who have been in Malaysia for several generations are fluent in the Malay language. Yet, the discriminatory behaviour towards non-citizens may be embedded within deeper undercurrents of xenophobia. This former student shared that she experienced bullying from her local classmates and was discriminated against by teachers at a public school because of her refugee status, despite sharing the Malaysian identity with no perceived language barrier, as she grew up in Malaysia.

*“Because I was growing up in a Malaysian community. I know their culture. I told you I have an identity crisis. I felt that I am one of the Malaysian students. Even though they know I can speak Malay, and I ‘plus-minus’ behave like one of them, but still, they know that I’m from Myanmar and my parents are from Myanmar. So, they call me ‘Budak Burma’ [Burmese Kid], and they do not want to come near me. They feel that I am smelly, that I am very different. Even though early in the morning, I come to school, just because I want to sit right in front of the teachers so that I can listen to everything. Somehow the teachers will ask me to sit at the back, right in the corner where nobody sees me. All of them call me ‘Budak Burma’ [Burmese Kid]. None of them wants to be friends with me until I was classified as an athlete.” FS-01*

This participant also shared that she experienced an identity crisis, as she felt Malaysian yet was identified as foreign by schoolmates and teachers at school. She informed that some Rohingya children would identify themselves as ‘Mamak’ (Malaysian of Indian Muslim heritage) in an attempt to better integrate with the locals and prevent bullying.

Another former student recalled that complaints of bullying by local classmates at a public school were not addressed by teachers and this experience led to her search for alternative learning centres.

*“And people just do not like you, and you do not feel you belong there. When you complain… the teachers do not take your complaints [seriously]. They kind of do not pay attention to you. And yeah, it kind of made my confidence [level] very low and my education was not good. So, then my dad found out [about] the X refugee school where a lot of refugee kids go to school. And in that school, there were a lot of Burmese students… Rohingya students. So, I feel more comfortable there. Even though the education was not formal, and we do not have proper teachers, proper syllabus, or proper books, but I feel more belong[ing] there, you know? People do not bully me.” FS—02*

Study participants reported better integration and a sense of belonging at learning centres hosting refugee or non-citizen children from similar backgrounds.

*Gender norms*, *culture*, *and language*. Most learning centres in Malaysia cater to children from diverse backgrounds. Interviewees share that although children may initially experience culture shock, these issues were usually addressed directly by education providers and easily resolved.

*“And of course*, *there was this struggle that we had at first between the different cultures coming together and like seeing the kids being shocked by something that the other kids do*. *And you know*, *like culture in some [communities]*, *like the Somali culture*, *the very famous thing is that all the female students need to have their head covered (hijab) regardless of age*. *Like you could have a 3-year-old having her hair covered*. *And that is not the same for other cultures*, *and that was one of the first things that we had to kind of sort out between children*.*”* FS– 07

Commitment towards education may also be related to the culture and background of the children. Participants explained that Rohingya children, in particular, would drop out of school at the post-primary level for different reasons according to gender, whereby the boys would enter the workforce, while Rohingya girls would stay home after puberty due to conservative community norms. This is in contrast with more recent Middle Eastern refugees that tend to be from a higher socioeconomic background and are more likely to prioritise education for all children irrespective of gender.

*“Okay*, *so for your information the enrolment in our schools*, *we have more students in lower primary compared to the upper primary*. *Meaning that for primary one/two/three*, *there are a lot of students coming to school*. *However*, *starting from primary four/five/six*, *some students will drop out*. *And this is a normal trend for the Rohingya community*, *usually based on several reasons*. *Mainly for boys*, *they have to stop schooling because they have to work to support the family*. *And for girls*, *the cases (are) usually when they reach puberty—there are some parents who are very conservative*,*(and) they do not allow children to go out freely*. *So*, *when they(girls) reach puberty and when they are at the age of a teenager… their parents would ask them to stop school and just help their mothers to do house chores in daily life*.*”* EP-05

Gender norms are not generalisable between communities. However, respondents affirmed that in most communities there isn’t gender bias in terms of educational access at primary levels, wherein boys’ and girls’ participation in primary education is near equal.

*“This is quite interesting in a way because when we look at the student data that we’ve been collecting over the years*, *gender parity is pretty good*. *Uh*, *like at primary level age*, *it is quite equal*. *However*, *when you get to the secondary level*, *there is a little bit of imbalance but then still*, *because boys were being pulled out and forced to work*, *while girls were being pulled out and forced to stay at home or forced into a minor marriage*, *so both genders have their challenges*, *you know*.*”* POL-03

Education providers informed that communication is the biggest challenge faced by children when first joining a learning centre. Although most children are unfamiliar with the English language when they start schooling, some education providers noted that children tend to pick up the language easily, which then becomes the unifying factor among different non-citizen communities. Refugee learning centres often preferred having the English language as the medium of instruction as it is a significant international language. This education provider explained that having good English language skills is often a necessary advantage during the resettlement process.

*“We have to teach them in English*. *The medium of instruction is in English*, *not in Malay*, *because they cannot use it [the Malay language] when they go [back] to their home country [or another country for resettlement]”* EP-03

Newly arrived refugees are usually only proficient in their mother tongues and learn English as it improves prospects of education, employment, and resettlement. However, the stateless, undocumented, and migrant children in Sabah, and the Rohingya children prioritise learning the Malay language instead.

#### Community and educational institutions

*Information provision and signposting*. Community organisers and former students shared that information on education options were not easily available. Newly arriving refugees would seek advice from community members and neighbours for suggestions on available learning centres or madrassahs located nearby. In our interviewees’ experience, this resulted in considerable uncertainty.

*“Alright, so when I came to Malaysia in 2014 –to be honest, they were no information and no resources that we know of where we could go for help, particularly for education. So, even the [available] Learning Centres we were not familiar [with] because in 2014 they were–very limited number of refugees… in Malaysia compared to 2020.” FS-04*

Although the UNHCR has a database of learning centres available to refugees in Peninsular Malaysia, it is unclear how widely this information is currently being shared and utilised among the community.

*Quality of teaching staff*. Most learning centres are unable to hire qualified teachers due to funding limitations and rely mainly on volunteers and teachers from the community. Former students interviewed informed that teachers were frequently absent from class and that there was a high turnover of the teaching staff at learning centres. This participant explained that this may be partially explained by the low-paying teaching positions and the reliance on volunteer teachers at learning centres.

*“The school needed a lot of improvements*, *in terms of quality of education and the teachers*. *You know*, *a lot of teachers were absent all of the time*! *And children go there*, *basically*, *just to play and have fun*, *not really to receive a proper education*. *I mean*, *I totally understand*, *because these teachers don’t get paid enough*. *You know*, *they get paid very little*, *very small amounts every month*. *So*, *I also understand that*, *if they were to be absent it was because they were considered volunteers*. *But you know*, *volunteers come and go*, *they do not stay that long*.*”* FS-03

According to study participants, volunteer teachers are usually well-intentioned Malaysians or expatriates who volunteer according to their availability. Volunteers were described as inconsistent, as they were unpaid, unable to commit to regular teaching schedules, nor trained in teaching a fixed syllabus. Former students described this lack of consistency experienced in learning centres as ‘confusing’ for learners, even though the students did gain some knowledge by meeting new people.

*“To be very honest*. *You could be confused*. *Because you have to give them [the volunteers] time… and they just come and go*. *And the other volunteers come and then—something different [will be taught]*. *So*, *it was confusing*. *It is interesting because we were able to meet people*… *we were able to gain from their knowledge*. *But at the same time*, *it was confusing as well*, *because you know they come and teach ‘this’*, *and they come and teach ‘that’*.*”* FS-02

Another former student described being taught certain basic lessons, like recognising alphabets and numbers, repeatedly by different volunteer teachers.

Teachers from the non-citizen communities may hold degrees or diplomas but generally lack teaching qualifications or experience. Consequently, learning centres conduct teachers’ training either internally or with help from partners at UNHCR or private international schools. The Indonesian Consulate supports a few estate-based learning centres by supplying qualified teachers and adopting the Indonesian syllabus, as observed in Sabah.

This education provider pointed out that the inability to hire qualified local teachers affects the learning centres’ ability to deliver education, especially at the secondary level. Instead, this gap will be filled by individuals from the community that are likely unqualified.

*“We do not have Senior High; we do not go that far*. *The reason is that my teachers are not really qualified*. *We are not allowed to employ qualified Malaysian teachers*. *So*, *I employ anyone in the area who wants to be a teacher and then we train them*. *That’s my job*, *training these people (unqualified teachers)”*. EP-04

Individuals who are willing to teach are often refugees or asylum seekers from countries where English proficiency is not emphasised. This is a setback in capacity building as competency in the English language is key in the education of refugees and successful integration in the transit or country of resettlement.

*“We have teachers who are also from the refugee community themselves*. *So*, *all those teachers in our school are either asylum seekers or refugees*. *Language is a problem because for many of these people that we have in school (teachers)*, *the countries that they come from do not speak English*, *and English is not a second language or a third language and it is not a requirement in some of the countries*. *For example*, *I had one teacher that told me that she did not need to know English when she lived in Syria*. *She did not have any need to learn how to communicate in English*. *But now that she is here*, *she has no choice*, *right*? *So many of my teachers also learn the language when they came here*.*”* EP-03

*Syllabus and accreditation*. In general, study participants commented on the lack of structure in the provision of education for non-citizens with irregular status. Participants explained that the lack of framework for alternative education in Malaysia stems from the lack of rights for refugees, asylum-seekers, undocumented and stateless persons.

Learning centres are not allowed to use the Malaysian national syllabus for instruction, yet many centres use syllabi based on Malaysia’s or other countries’ national curricula. Some learning centres used curricula which are non-formalised amalgamations of different syllabi based on available resources and expertise. The Malaysian national syllabus is commonly used by learning centres, as textbooks and other materials are easily available, and the instructors are familiar with the material.

*“Our main goal*, *is first we want to solve the literacy and numeracy problems of these marginalised children*. *So*, *we did a little bit of research with existing preschool publications*, *for example*, *the ‘Bacalah Anakku’*, *the ‘Pelangi’ publications and others*. *We feel that the most complete and most suitable syllabus to use at any level and to solve this literacy and numeracy problem… the best syllabus now is from the KPM [Ministry of Education (MOE)*, *Malaysia] itself*. *So now we use the syllabus from the MOE*.” EP-02 (translated from Bahasa Malaysia)

Learning centres may adopt assessments equivalent to the government school-leaving examinations (e.g., IGCSE, fifth form or year 11), but since these assessments are often unaccredited, their value remains abstract. Refugees interviewed shared that not having internationally recognised school leaving certificates effects their prospects in countries of resettlement, where resettled refugees would have to start over their education journey.

*“Even if you go to learning centres that have been founded to help refugees and you do happen to graduate, it really doesn’t (matter)–I do not want to say it does not matter… (because education is important). However, you do not have anything to show for it. Once you get resettled in a different country—So, what will happen is you will just have to start over again. Since you do not have anything that is recognised… since there is no certificate or anything that shows that you have graduated high school.” FS-07*

Education providers realise the importance of obtaining recognisable school-leaving certificates for employability, further education, and resettlement and that aiming to achieve basic literacy and numeracy alone may be insufficient in the long term.

An interviewee commented that refugee learning centres were increasingly opting for IGCSE (The British O’ Levels) at the secondary level as international qualifications would be beneficial for resettlement or return to home countries.

*“However, when you look at the curriculum and syllabus at the primary level, 90 to 95% of curriculum and syllabus worldwide are the same anyway. So, it is of little matter which curriculum and syllabus they choose. It is only when they get to the secondary level then it has a greater bearing—the learning centres are opting for IGCSE because it is internationally recognised. So, whether the refugee student remains in Malaysia, that paper is [still] recognised by tertiary institutions.” POL-03*

Education providers informed that some learning centres offer vocational training as an option for older students who are unable to pursue the conventional academic route. Vocational training aims to develop income-generating skills to improve employability and provide more options for school leavers.

*“For example*, *we have students who are over the age to be enrolled in secondary school… because when they enrol in our school*, *they are already a teenager… So*, *they do not seem keen to continue studying*. *So*, *there is a vocational class*. *We want to give more options to our students to continue their education*, *not only academically*, *but as well as with life skills*. *We hope that from our vocational class when they learn how to sew–for example*, *the Baju Kurung (national dress) … Perhaps it helps develop additional skills that can be used as a source of income*. *So*, *our long-term target is that they gain skills that they can use to generate income to help their families financially*.*”* EP-05

*Physical facilities*. Participants informed that since resources are limited, learning centres are often located in premises like shop-lots, empty houses, and other spaces that are available for rent, or borrowed or donated by individuals or societies. Learning centres are seldom located in buildings purpose-built as schools and generally lack the space and facilities compared to public schools. This former student shares that community centres for refugees are utilised as temporary learning centres for children.

*“These refugee families live in different areas…Refugee communities do not all live in just one area. So, each area would have at least one or two community centres that serve specific communities. These community centres are turned into Learning Centres for the children. The space is very small and there are so many children, and it doesn’t fit. At some of these schools, they have to sit on the ground because there’s no space to even put chairs and tables. Facilities are very poor. And there are many, many children. The demands are high, the school cannot do much because there is no funding.” FS-03*

The lack of facilities and digital infrastructure was especially challenging during the COVID-19 pandemic and lockdowns when learning centres were forced to close and struggled to convert lessons into a digital format.

Participants informed us that registration of learning centres is almost impossible in Sabah, where the state authorities only recognise learning centres in plantation estates that are supported by the Indonesian embassy. Learning centres that are not estate-based (located in towns) generally lack resources, have poor infrastructure and are at constant risk of closure. Education providers shared experiences of being forced to rent buildings that do not offer protection from the weather and are thus unsuitable for teaching and learning.

*“The class may be cancelled depending on the weather condition “So we do not have enough space, we do not have proper tables and chairs… when it rained the class have to be cancelled because we do not have proper space, right? And then sometimes it was too hot, and the kids are very uncomfortable, yeah.” EP-10*

Study participants spoke of the unmet demand for education among marginalised communities in Sabah. This participant explained that civil society organisations were limited by their lack of resources and inability to afford space for learning centres.

*“First*, *we must understand that XXX is not able to accommodate all these children*. *They are too many of them*. *If you follow the latest statistics from Sabah*, *there are more than 100 thousand children without documents*. *It is impossible for any NGO to be able to help this many [children]*. *That is why we demand that it is the responsibility of the government to intervene*. *So*, *we can only set up our space for 30 people [and] that too*… *we are already in the living room*. *So*, *we could not afford to cater to all of them*, *so we put up some criteria*.*”* EP-11

He explained further that resource limitations forced learning centres to restrict classes to older children, trusting that they would be able to instruct their younger siblings at home.

*Fees*, *funding*, *and operational costs*. Many learning centres prioritise educating children and provide subsidised school fees. Education providers explained that the fees charged were nominal and were considered commitment fees for students and families to appreciate the value of education.

*"Every day we support the children with transportation going and back from school*, *as well as light meals during the break*. *So*, *it’s barely like one Ringgit a day in fees to cover the meals and transportation*. *So*, *that’s how we try to justify to the parents*, *the importance of the school fee*. *Although we have financial support from UNHCR and XXX*, *but it’s not enough to cover our daily expenses of schools*. *For example*, *the meals and the stationary*, *the utilities*, *and the maintenance of the schools*. *So*, *the school fees actually do play a crucial role as well*, *but we have to work hard to explain to parents how they should be responsible in helping children to get better quality education*.*"* EP-05

Education providers argued that school fees were important, as they were used to pay for school meals and transportation, and even to supplement the operational costs of the learning centres.

As the fees collected are minimal, education operators must rely on financial support from local and international donors, including individuals, community members, NGOs, philanthropic organisations, corporate bodies, and others. Having a steady income stream is important for the sustainability of operations. Education providers explained that funding sources are often inconsistent, placing the continuity of education programmes under considerable uncertainty.

*“Even with the help of donors*, *the funding is not something that is consistent*. *It is only when we have the funding is when we can move*. *Okay*, *like last year*, *we lost a funder–we did not [exactly] lose a funder–We had a funder who funded our secondary education for six years*. *But because of their own policies*, *they only fund for a two-year cycle or maybe a three-year cycle*, *so then they have to move on to some other organisation*. *So*, *we thought that we would lose our Secondary School*. *But we managed to secure some funding from some other people and we were able to move the education programme forward*.*”* EP-03

This respondent commented on the inequalities observed among refugee learning centres, where there are only a few well-funded centres, which contrasts with most learning centres which are community-run and less supported. The better-resourced learning centres have been described as similar to private international schools and have better facilities, a higher quality of teachers, the use of international syllabi and access to accredited examinations.

*“There are few, very few–three or four refugee schools that are doing very, very well. They are well-funded. They are not run by the (refugee)community, but they are run by Malaysians or NGO-run organisations. And they are well funded, which means [that] they have full-time teachers or very qualified [teachers], they have all the facilities they need, and [students may take international] examinations [for] qualifications at these facilities. And not only that–but the curriculum is verified and everything. For example, you have these four refugee schools that are–I would say–they are well funded. And they are way, way better compared to other refugee schools that do not even have funding to even pay the rent of the premises, do not even have funding to even buy books for their children. And so, there is huge inequality and a huge gap when it comes to children’s education.” FS-03*

Better-resourced centres are in high demand and have waiting lists for school enrolment, even though education providers informed us that they try to enrol as many students as they can.

*Diverse school readiness and educational backgrounds*. This education provider explained that non-citizen children may require additional preparation to adjust to learning environments considering their deprived backgrounds, delayed start of schooling and poor school readiness. She points out that public schools are not suitable for marginalised children, as the education system does not address poor school readiness.

*“I believe that children in this predicament should not go to government schools for the first two or three years of their educational lives. And my reasoning is that they come from a very poor, limited background of preparation for primary school[ling]. A Malaysian child is ready for school because his family have talked to him about school from when he was an infant. And he knows and the family knows the importance of education. They have books in their house. Their psyche, their whole being is ready for school. But our children are discriminated against–they know that they are discriminated against because the community discriminates against them. They have very low self-esteem and self-esteem is critical, absolutely critical for a child beginning in school to fit in and to get on and to have the courage and the feeling of ‘I can do it!’” EP- 04*

Study participants informed that teaching at learning centres was challenging as children were from different backgrounds and educational levels. Refugee children born in Malaysia and those recently arriving from their countries of origin have different competencies in languages and learning, and teachers need to adjust their delivery accordingly.

Unlike Malaysian public schools where age determines the student’s grade level, new students at learning centres would undergo placement tests or assessments to determine their literacy and educational levels before being assigned to a class. Some learning centres offer preparatory programmes for late learners and newcomers to help them catch up with their peers.

*“In certain countries*, *you will find the students a bit better educated than in other countries*. *And in all my grades*, *you will have this age range*, *because they all did not achieve the same levels [of education]*. *So*, *when they come in*, *we give them a placement test to place them*. *Because it’s difficult for teachers to address like all 7-year-olds in one class and then address at every level*, *you know*. *It is just simply difficult*. *So*, *we do the placement testing and place them in groups together*. *Those who we cannot*, *we put them in separate classes on the side*. *Like a kind of a special class to bring them up to speed*.*”* EP—03

At more established learning centres, students have the flexibility to advance rapidly to higher grades according to their achievement in placement assessments.

## Discussion

In this research, we aimed to understand the barriers and facilitators to educational access faced by marginalised non-citizen children in Malaysia, through in-depth interviews of multiple stakeholders. We uncovered detailed demand- and supply-side barriers at every stage of accessing education. What is striking is the level of similarity of educational barriers that cut across diverse non-citizen groups, potentially signifying “major, critical bottlenecks” to education access [34]. Our analysis proposes that this shared experience of educational exclusion can partly be attributed to macro policies affecting all groups in this study. Besides the direct impact of education policies, other interconnected policies related to legal status (e.g., citizenship, migration, asylum, regularisation) and employment exerted a considerable indirect impact on marginalised non-citizen children’s education. Our findings on several specific educational barriers and the centrality of legal status are similar to the wider literature on international migrant and refugee children’s access to education [35–37]. As for the indirect impact of employment policies, our findings illustrate how adults’ (lack of) right to work impacts children’s right to education. Without the right to work, marginalised non-citizens are forced to participate in low-wage, unsafe, and insecure jobs in the informal sector which puts them at risk of exploitation by employers [38–41]. Not only are many non-citizen families unable to afford schooling expenses, but children also face financial pressures to enter the informal workforce prematurely [42–44]. Moreover, schooling is sometimes discouraged as education does not improve employment prospects in this context. Children’s low educational attainment then impairs the livelihood prospects of the next generation. Thus, there is a strong case to register stateless and undocumented persons and provide the ‘Right to Work’ for refugees, to prevent child labour and break the vicious cycle of poverty.

A theme that cuts across socio-ecological levels is discrimination, which was present from the interpersonal level (e.g., bullying and social ‘othering’ from local peers and prejudicial treatment by local teachers), to the institutional level (e.g., excluded from full participation in the economic, social, and political life of mainstream society). Discrimination also threatens safety during travel given the dangers of harassment by authorities and abuse by citizens [45, 46], which may partially explain parents’ reluctance towards schooling. However, our findings suggest that discrimination due to cultural differences is less of a concern at learning centres that cater to marginalised children, as teachers can recognise and establish countermeasures early. Discrimination is related to “relational” dimensions of educational inclusion, which refers to the personal sense of belonging and group-level social cohesion between non-citizens and host populations [37, 47, 48]. As seen in other settings, experiencing discrimination and lacking a sense of belonging in school may affect a child’s academic performance, damage physical and mental health, and lead to poor psychosocial adaption and substance abuse [49–53]. Thus, education systems and schools should celebrate student diversity and reduce discrimination through inclusive education [54, 55]. It is widely recognised that developing a positive ethnic and cultural identity in school and other settings may also buffer the effects of teacher and peer discrimination [56–60]. Towards this end, teacher training modules on anti-discrimination, cultural and gender sensitivity, and language development should be created for public schools and learning centres.

Malaysia has made tremendous progress in closing education gaps across urban-rural areas, socio-economic class, and gender [61]. For non-citizen children who have fallen through the cracks, non-state actors have shown great commitment, adaptability, and ingenuity to meet this education gap. Yet, it is important to recognise the limited scale and impact that can be achieved by an under-funded, fragmented, parallel education system operating under unfavourable socio-legal conditions. Thus, recent government initiatives and collaborations to expand educational access for out-of-school children, such as undocumented Malaysians and migrant children in Sabah, is a welcome move, and one that aligns with broader aspirations of the Malaysian education system towards greater access, quality, equity, unity and efficiency [61]. To overcome the remaining challenges towards universal basic education, it is paramount to leverage this momentum and increase collaboration to develop solutions.

Besides the facilitators we identified, broader refugee education literature offers strategies that can be adapted to different non-citizen groups in Malaysia. Globally, there has been a strategic shift from parallel, temporary education systems towards an “inclusive national education system” strategy for refugee children, which involves working closely with UNHCR, UNICEF and Ministries of Education to strengthen national education systems to benefit host communities and refugees alike. This is the key recommendation based on an extensive review of global evidence in 2011 [62] and is better aligned with the realities of contemporary migration patterns. Specifically, the up to threefold-increase in the average duration of displacement since the 1990s means that temporary education systems are no longer serving refugee children whose entire education lifecycle may take place in exile [63–65]; growing proportions of urban versus camp-based refugees worldwide means that refugee-only schools are increasingly impractical in urban areas where host communities also reside [66]; the unpredictability and persistent under-funding for refugee education means that parallel education systems often struggle to deliver quality education and are unsustainable in the long-term [67]. For these reasons (some of which are applicable to non-refugee, non-citizen children), this strategic shift towards an inclusive national education system has since been affirmed in the UNHCR Global Education Strategy 2012–2016 [68], UNHCR Refugee Education 2030 strategy [69], Global Compact for Refugees 2016 [70], and the 2017 Djibouti Plan of Action, which represents eight member states’ commitment towards this goal [71, 72].

A comprehensive review of best practices towards inclusive national education systems is beyond our study scope. However, a 14-country analysis provides insights into emerging models: (1) *shared space* (non-citizens and citizens learn in the same physical classroom), and (2) *separate space* that can be divided into (2a) *geographically separate* (non-citizens and citizens attend different schools) and (2b) *temporally separate* (non-citizens and citizens attend the same school at different times, or “shift systems”) [37]. The analysis listed promising practices which considered “relational” and “structural” dimensions of inclusion (e.g., access to nationally accredited certifications, the use of national curriculum, teachers, and language of instruction). The UNHCR Refugee Education 2030 also details other best practices globally (e.g., accelerated learning programmes) [69].

Access to basic education is a human right. Nevertheless, policy stakeholders will likely have reservations about the financial costs and impact on the national economy, particularly where the educational needs of out-of-school citizen children remain unmet. Yet, there is substantial evidence that education consistently yields returns to investment, averaging at a 9% rate of return to one additional year of schooling over six decades [73]. In an economic analysis of seven Southeast Asian countries (Malaysia excluded), *not* reducing the number of out-of-school children in primary schools is estimated to forfeit between 0.1%-4% of GDP due to undereducated workers [74]. In other words, there is an economic cost for inaction. Conversely, achieving universal primary enrolment is estimated to generate economic gains *exceeding* increased public spending needed to enrol out-of-school children in primary school (e.g., building new schools) [74]. The report concluded with strong *equity and efficiency* arguments in favour of expanding quality primary education to out-of-school children. In another global costing study for inclusive refugee education, it is estimated that the cohort-average annual cost for refugee education is under 2.5% of Malaysia’s public expenditure on primary and secondary education [75], concluding that “‘what it would take’ [for inclusive refugee education] is not out of reach of the collective efforts of the international community and host governments” [75]. Governments do not have to shoulder the financing burdens alone. There has been a growing interest in “innovative financing” mechanisms to share financial responsibilities across public sectors, private sectors, and communities by (1) raising new resources from non-traditional funding sources, and (2) using existing resources more efficiently [76]. For instance, ‘public-private hybrid schools’ and co-funding models [77] have been observed in our study, where private oil palm companies contribute land and infrastructure, government(s) contribute qualified teachers, and communities themselves contribute in-kind support by volunteering to teach or build classrooms. A wider menu of innovative financing mechanisms has been documented elsewhere [76, 78].

Economic and rights-based arguments aside, we acknowledge remaining tensions regarding immigration concerns of providing education to non-citizen children. Yet, as alluded to in our study, providing education for all children is not necessarily a zero-sum game for citizen children. For instance, our study that began by examining the educational needs of non-citizen children brought to light a group of undocumented Malaysians facing similar barriers [30]; some education providers in our sample have been catering to undocumented Malaysians alongside non-citizen children; facilitators, considerations, and innovations to expand educational access among non-citizen children are transferrable to out-of-school citizen children to a certain extent. Although there remains uncertainty on how to resolve these dilemmas, what is clear is that inaction is not an option.

Therefore, we propose the gradual inclusion of non-citizen children into the national education system, starting with primary schools, as this is the only viable option for education for all. We hope that our study which has identified *who* and *how* groups of non-citizen children have fallen through the cracks will provide a way forward into constructive dialogues between state and non-state stakeholders, involving education experts in all aspects of planning. Suggestions informed by this study include designing catch-up programmes for varied school readiness and language needs, sensitising local teachers and students to policy changes, promoting diversity and inclusion, adopting trauma-informed approaches for refugee students, establishing accreditation and pathways for education continuity, and eventually, decent employment options. Until this is possible, the Malaysian government should support learning centres by allowing unhindered operation, the use of the national syllabus, providing avenues for teachers’ training, access to subsidised books and other materials, and financial support, especially for smaller learning centres serving marginalised communities.

### Strengths and limitations

Unless otherwise specified, barriers and facilitators identified here are largely generalisable to the different groups of non-citizen children in Malaysia. Yet, a detailed analysis of individual marginalised non-citizen groups is likely to yield different levels of education access and outcomes due to the intersectionality of population characteristics, which brings with it layers of privilege or marginalisation. With this in mind, we have established our interview approach to understanding participants’ individual endeavours and experiences in education access. We were conscious to avoid establishing any views of our own. Future research would benefit from examining the influence of gender and disability on education access within marginalised non-citizen groups, in line with goals for inclusive education.

Due to its qualitative nature, study findings cannot be readily generalised to other settings with different policies, provisions, and protection for non-citizen children. Yet, this study is unique as it allows the understanding and identification of major barriers to education access faced by marginalised non-citizen children, families, and communities while highlighting strategies and adaptations by the community and educational institutions that serve as facilitators.

## Conclusion

Education is an enabling right, instrumental for social mobility and poverty eradication. To this end, we systematically explored the challenges faced in achieving educational access for marginalised non-citizen children in an upper-middle-income country. Policy recommendations include the gradual inclusion of all children in public schools, state recognition and support of learning centres, and the realisation of the ‘Right to Work’ for refugees, asylum-seekers and stateless peoples.

## Supporting information

S1 File (PDF)
